# Use of a modified version of the switching verbal fluency test for the assessment of cognitive flexibility

**DOI:** 10.1590/1980-57642015DN93000008

**Published:** 2015

**Authors:** Jonas Jardim de Paula, Gabrielle Chequer de Castro Paiva, Danielle de Souza Costa

**Affiliations:** 1 Departamento de Psicologia, Faculdade de Ciências Médicas de Minas Gerais, Belo Horizonte MG, Brasil.; 2 Laboratório de Experimentação em Psicologia e Neuropsicologia (Labep_neuro), Belo Horizonte MG, Brasil.; 3 Instituto Nacional de Ciência e Tecnologia em Medicina Molecular, Universidade Federal de Minas Gerais, Belo Horizonte MG, Brasil.

**Keywords:** neuropsychological assessment, psychological assessment, neuropsychological tests, cognitive functions, executive functions

## Abstract

**Objective:**

Verbal fluency tests are widely used for the assessment of executive functions. However, traditional versions of the test depend on several cognitive factors beyond these components. The aim of this study was to evaluate the associations of a modified version of the verbal fluency with specific measures of executive functions.

**Methods:**

Sixty adults were evaluated using traditional versions of verbal fluency (animals/fruits) and a modified condition where subjects must switch between animals and fruits. Processing speed, semantic abilities, psychiatric symptoms and executive functions were also assessed.

**Results:**

Partial correlations between the verbal fluency tests and measures of executive functions, controlled for demographic, cognitive and psychiatric symptoms, suggest that cognitive flexibility has 9% shared variance with the verbal fluency test – category animals, 2 % with category fruits, 8% with total words in switching condition, and 20% with total correct word-pairs produced in switching condition. The other aspects of executive functions during the task had shared variance of between 1% and 7% with the verbal fluency tasks.

**Conclusion:**

The results suggest that correct word-pairs produced in switching verbal fluency may be a more specific measure for evaluating cognitive flexibility compared to other versions of verbal fluency.

## Abstract (pt)

**Objetivo:**

Os testes de fluência verbal são comumente adotados para a avaliação cognitiva de pacientes com transtornos mentais ou doenças neurológicas. Embora muitas vezes utilizado na avaliação das funções executivas, sobretudo flexibilidade cognitiva, versões tradicionais do teste dependem de diversos fatores cognitivos além desses componentes. O objetivo do presente estudo é avaliar as associações de uma versão adaptada do teste de fluência verbal com medidas específicas de funções executivas.

**Métodos:**

Avaliamos sessenta adultos com versões tradicionais da fluência verbal (animais/frutas) e uma condição modificada onde o sujeito deve alternar entre animais e frutas. Avaliamos também a velocidade de processamento, habilidades semânticas, sintomas psiquiátricos e aos três componentes centrais das funções executivas (controle inibitório, memória de trabalho e flexibilidade cognitiva).

**Resultados:**

As correlações parciais entre os testes de fluência verbal e as medidas de funções executivas, controladas por fatores demográficos, cognitivos e pelos sintomas psiquiátricos, sugere que a flexibilidade cognitiva apresenta 9% de variância compartilhada com o teste de fluência verbal categoria animais, 2% com a categoria frutas, 8% com o total de palavras na fluência verbal alternada, e 20% com total de pares corretos produzidos na fluência verbal alternada. Os demais aspectos das funções executivas durante a tarefa apresentaram variância compartilhada entre 1% e 7% com as tarefas de fluência verbal.

**Conclusão:**

Os dados sugerem que o total de pares produzidos no teste de fluência verbal alternada pode ser uma medida mais especifica para a avaliação da flexibilidade cognitiva quando comparado a outras versões da fluência verbal.

## INTRODUCTION

Executive functions are top-down cognitive processes related to the control of behavior and other cognitive functions.^1^ In a recent review, Diamond^2^ proposed a model of executive functions characterized by three basic processes: working memory – related to mental manipulation of information; inhibitory control – related to the inhibition of thought, behavior and attentional distractors; and cognitive flexibility – related to switching between tasks or actions and creative thinking. The assessment of these functions is one of the most important points of neuropsychological assessment, since the integrity of the executive function is closely linked to functional outcomes such as school performance,^3^ labor capacity,^4^ activities of daily living^5^ and quality of life.^6^

Diamond^2^ suggests verbal fluency tasks as a useful measure for the assessment of cognitive flexibility. Verbal fluency involves the production of words, generally associated with a category (such as animals, fruit or parts of the body) or beginning with a particular letter or sound (such as the letter "F" "A" or "S"). Verbal fluency tasks are widely adopted in the evaluation of executive functions and language.^7^ Regarding anatomical and clinical correlates, these tasks are commonly associated with prefrontal regions of the left hemisphere in studies involving functional neuroimaging^8^ and brain-damaged patients.^9^

A limitation of these tasks is their strong association with several cognitive domains. In the Brazilian population, verbal fluency tests were associated with semantic knowledge,^10^ executive functions and processing speed,^11^ intelligence and attention^12^ as well as sociodemographic aspects including age, education and gender.^13^ In this context, Diamond^2^ suggests modifications for tests of verbal fluency to increase their specificity for the assessment of cognitive flexibility, including a switching condition between different items of information (e.g.: objects and food). This form of administration involve the dynamic switching between two or more different sets of information, requiring greater recruitment of the cognitive flexibility function. The neuropsychological battery *Delis-Kaplan Executive Function System*,^13^ provides an alternate version of the verbal fluency test, in which the subject must switch between names of fruits and furniture items, in addition to traditional versions involving the letters M, A and S, and the categories animals and boys' names.^14^ The procedure shows evidence of validity for the assessment of clinical groups with frontal lobe dysfunction, including patients with focal brain lesions,^15^ autism^16^ and traumatic brain injury.^14^

We propose a modified version of the switching verbal fluency test. The procedure involves two categories commonly used in Brazilian studies of verbal fluency: animals and fruit.^10,18^ The objective was to compare traditional versions of the semantic verbal fluency test against a modified version of the switching fluency test focusing on its associations with different aspects of executive functions.

## METHODS

**Participants.** The study included 60 adults with a mean age of 27.92±11.35 years. The participants comprised a convenience sample recruited locally by the authors. Inclusion criteria were: absence of self-reported sensory or motor impairments that might influence the neuropsychological tasks; having Brazilian Portuguese as first language; aged 18 years or older; and signed the informed consent form for the study. This study is part of a larger project related to the adaptation of the inhibitory control test for the Brazilian population and approved by the local research ethics committee. The demographic profile of the sample is shown in Table 1.

**Table 1:** Description of participants (N=60).

Age	Mean	27.92
	Standard deviation	11.35
Sex	Male	32%
	Female	68%
Formal education	Primary	5%
	Middle	58%
	College	37%
Occupation	Study	32%
	Work	32%
	Study and work	30%
	Neither study nor work	6%
Socioeconomic status^1^	A	22%
	B	47%
	C	28%
	D-E	3%
Self-reported diagnosis of psychiatric disorder or neurological disease	No	80%
	Yes	20%
Use of psychotropic pharmacotherapy	No	74%
	Yes	26%
Symptoms of Depression/Anxiety^2^	No	85%
	Yes	15%
Symptoms of ADHD^3^	No	83%
	Yes	17%

1 Classified according to Critério Brasil (ABEP, 2015);

2 Based on the Self-Reported Questionnaire 20 (Mari & Williams, 1986);

3 Based on the Adult Self-Reported Scale 18 (Mattos et al., 2006).

### Neuropsychological assessment

**Verbal fluency tests.** The semantic verbal fluency tests with the categories Animals and Fruit were used in this study. The semantic version was selected as opposed to the lexical/phonemic versions due to the educational heterogeneity of the local population, which sometimes limits the use of instruments that require knowledge acquired through higher formal education. A 60-second limit for responses within each category was allowed. Higher scores represent better performance. The following instructions were used:

"*In the test that follows, I want you to say words for a certain category. For example, if the category is "colors" you must give me names of colors like blue, yellow, red, green ... variations of the same color don't count, such as light blue, dark blue, jasmine blue... okay? I will set a time and want you to say the words until I ask you to stop. In the first part, say animal names. Tell me all the animals that you can remember. You can start. (...) Now, tell me the names of all fruit you know. You can start now*."

**Modified switching verbal fluency test.** Immediately after the application of semantic verbal fluency tests, the experimental task was applied. In this condition, the participant is expected to alternately say the name of an animal followed by the name of a fruit. It was emphasized that the names of animals and fruit given earlier in the traditional version of the task could be used. It was specifically explained to the subject that they could say any animal and any fruit, because in an earlier unpublished pilot study participants had tried to spontaneously produce associations between animal and fruit (like "monkey and banana"). The previously tested categories were used, a procedure that differs to the most used switching verbal fluency test.^13^ This modification aims to minimize memory retrieval processes during the task, allowing a more circumscribed process of cognitive flexibility. In the task, the total number of words produced within 60 seconds is recorded, the number of correct word-pairs formed during the same timeframe (each pair is composed by an animal followed by a fruit but the use of a fruit followed by an animal was also scored as correct) and total errors made by the subject. Higher scores represent better performance. Application instructions and correction used in this study were as follows:

"*To finish this word test we will now mix the two categories. I want you to tell me the name of an animal, and then the name of a fruit. The name of an animal, the name of a fruit. You will switch between those two categories, giving one and then the other. You can repeat the words you said on the two previous tests, bust should avoid repetitions in this task. It can be any animal, any fruit, they do not need to be related. You can start now*."

**Boston Naming Test.** This task is used as an estimate of semantic knowledge of the participants. In the present study, only the even items from the adapted version for the Brazilian context proposed by Mioto et al.^19^ were used. The total test score ranges from 0 to 30. Higher scores represent better performance.

**Trail Making Test.** This test is commonly used for the evaluation of attentional processes and cognitive flexibility. The Trail Making Test contains two components. The first part only demands simpler attentional processes – seeking numbers spread throughout a page, while the second involves a flexibility component requiring the participant to switch between numbers and letters during the test run. We used the original version proposed by Reitan^20^ validated for the Brazilian context.^21^ As a measure of cognitive flexibility, the ratio of time spent on the second part of the test and the time spent on the first part of the test (B / A) was used. Higher scores represent poorer performance.

**Five Digits Test.** This task is an attentional interference test (Stroop effect), whose stimuli involve numbers and quantities. The original version proposed by Sedó^22^ is currently being validated for the Brazilian population with preliminary results already published.^10,23^ Three steps of the task were used. First, the subject is introduced to 50 stimuli (rectangles) aligned in 5 rows and 10 columns containing one to five numeric symbols (the numbers 1 to 5) congruently (1; 2-2; 3-3-3; 4-4-4-4, 5-5-5-5-5). The second part involves the count of quantities ranging from 1 to 5 elements (asterisks) in each of the 50 stimuli (*, **, ***, ****, *****). The third part involves an interference condition in which the subject must count the Arabic numerals at each stimulus ignoring the name of the digits (incongruously) (2; 3-3; 1-1-1; 5-5-5-5; 4-4-4-4-4). The time in seconds to perform each step was recorded. Based on the runtime of each part of the test, two variables were created. The first is the participant's processing speed, and was calculated by summing the reading and counting times. The second is the interference control, a component of inhibitory control, calculated based on the time difference between the third and first parts of the test. Higher scores represent poorer performance.

**Digit span task.** This is a classic test for the evaluation of short-term memory and executive components of working memory. The test consists of the repetition of a growing sequence of digits, initially in the forward and then in backward orders. In the present study, a score comprising the sum of forward and backward versions of the task was used, applied and corrected according to Kessels et al.,^24^ previously validated in the Brazilian context.^18^

**Statistical analysis.** Pearson's partial correlations were used to analyze the pattern of associations between cognitive measures. The association of verbal fluency tests (animals, fruit and switching) with measures of executive functions (cognitive flexibility, working memory and inhibitory control) were controlled for the effects of age, education, sex, processing speed, semantic knowledge and psychiatric symptoms. The shared variance was calculated by the coefficient of determination (r^2^). The statistical procedures were performed using SPSS 20.0 software.

## RESULTS

Participants' description, performance on neuropsychological measures and results from the assessment of psychiatric symptoms are shown in Table 2. With respect to verbal fluency, the modified switching version exhibited an intermediate value (18) between the mean of words produced in animals (20) and fruit (16). The mean of word-pairs produced correctly (8) in the alternate version was slightly less than half the total of words produced under the same conditions for the traditional version. Although no formal screening for cognitive impairment was conducted as an inclusion criteria, no participants in the study scored below the 2^nd^ percentile (approximately -2 standard deviations) when compared to a reference sample (n=260) stratified by education on the verbal fluency task (animals).^7^

**Table 2:** Participant performance on neuropsychological tests and self-reported psychiatric symptoms.

Domain	Measure	M	SD	Min	Max
Verbal fluency	Animals	20.17	5.98	10	42
	Fruit	16.10	3.75	9	28
	Switching (words)	18.17	3.76	10	30
	Switching (word-pairs)	8.22	1.97	3	13
Semantic knowledge	Boston Naming Test (30 items)	26.03	3.21	14	30
Processing speed	FDT Reading + Counting	48.10	12.21	24	80
Cognitive flexibility	Trail Making Test B/A	2.27	0.81	0.92	5.06
Working memory	Digit Span Forward+Backward	84.13	35.90	18	173
Inhibitory control	FDT Inhibition	15.97	7.57	–3	34
Psychiatric symptoms	SRQ-20 (Depression/Anxiety)	5.58	4.10	0	16
	ASRS-18 (Inattention)	15.58	5.27	7	25
	ASRS-18 (Hyperactivity)	14.37	6.46	0	30

M: Mean; SD: Standard-Deviation; Min: Minimum; Max: Maximum; VF: Verbal Fluency; FDT: Five Digits Test; SRQ-20: Self-Reported Questionnaire; ASRS-18: Adult Self-Report Scale.

**Figure 1:**
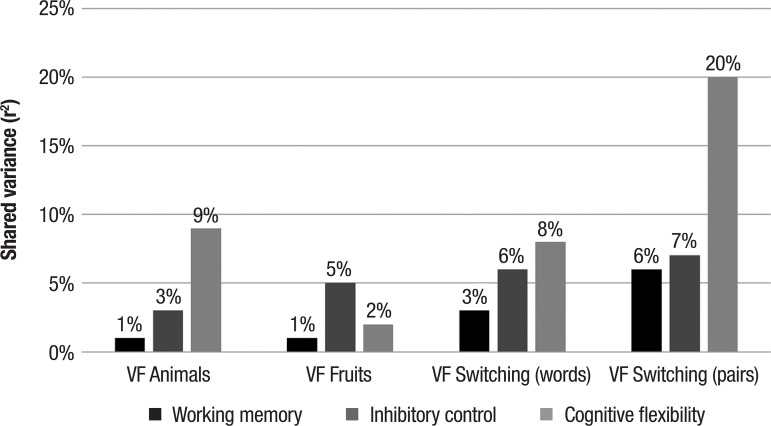
Shared variance between different verbal fluency tests and specific aspects of executive functions.

The correlations between verbal fluency tasks and executive functions, controlling for the effect of age, education, gender, psychiatric symptoms, semantic knowledge and processing speed are shown in Table 3. The shared variance between different versions of the verbal fluency test used in the study with the measures of executive functions is shown in Figure 1. The results of the association analysis suggest that the number of pairs produced in the adapted alternating verbal fluency test is more strongly associated with executive functions, particularly cognitive flexibility, than the traditional versions or the full words produced in the alternating version.

**Table 3:** Partial correlations between verbal fluency tests and different aspects of executive functions.

Test		1	2	3	4	5	6	7
1	VF Animals	1.000						
2	VF Fruits	0.348*	1.000					
3	VF Switching (words)	0.535**	0.536**	1.000				
4	VF Switching (word-pairs)	0.429**	0.432**	0.772**	1.000			
5	Cognitive Flexibility	–0.292*	–0.124	–0.280*	–0.445**	1.000		
6	Working Memory	–0.079	0.029	0.169	0.249	–0.293*	1.000	
7	Inhibitory Control	–0.162	–0.224	–0.352*	–0.270	–0.071	0.022	1.000

Controlled for age, education, sex, psychiatric symptoms, processing speed and semantic knowledge. VF: Verbal Fluency.

* p<0.05,

** p<0.01.

## DISCUSSION

The present study examined the contribution of different aspects of executive functions to performance on three tests of verbal fluency. The findings suggest that the correct word-pairs produced in the alternate version of verbal fluency, adapted in this study using the categories animals and fruit, was more strongly associated with executive functions than traditional versions of the test. As hypothesized, and according to that proposed in Diamond's^2^ model, the cognitive flexibility component showed the strongest association with verbal fluency tests.

Verbal fluency tests are widely used in the Brazilian context. According to a review,^28^ the test is the second-most-used for the evaluation of dementia in the country. Although the test provides an estimate of executive functioning, the traditional versions may be more related to language and processing speed than to executive functions properly.^29^ Accordingly, the use of a modified "switching" version of the task could increase specificity for the assessment of executive functions, especially cognitive flexibility. However, it is important to note that the shared variance of the modified switching version with traditional measure of cognitive flexibility proved only moderate (R^2^=20%) in this study.

There are few studies examining the construct validity of the alternating verbal fluency test, which limits the scope for interpretation of our results. Stolwyk and colleagues^29^ analyzed the association of different cognitive aspects with verbal fluency tests in samples of younger and older subjects. Their findings suggest that in younger participants, semantic retrieval processes (measured by timed picture naming) are be the main predictor of verbal fluency, while in older participants, crystallized intelligence was the main predictor. Henry & Phillips^30^ analyzed the cognitive predictors of a switching verbal fluency test and found significant effects of fluid intelligence and processing speed, but not of crystallized intelligence. Parkin, Walter & Hunkin^31^ found correlations of a version of alternating verbal fluency with the Wisconsin Card Sorting Test, a traditional measure of cognitive flexibility, even after statistical control for intelligence. Iudicello et al.^32^ found correlations of switching fluency with verbal working memory and semantic knowledge, but not with cognitive flexibility. Nutter-Ipham et al.^33^ conducted a study which analyzed the factor structure of different fluency tests, finding a decoupled factor for switching fluency compared to other fluency tests.^33^ The authors also found correlations between alternating verbal fluency and intelligence measures, semantic knowledge, processing speed and cognitive flexibility. These studies, however, relied on the common versions of switching verbal fluency, where the categories used were new to the task. We believe that using the same categories previously employed in the traditional verbal fluency tasks during the switching version may reduce demands on crystalized/semantic knowledge and retrieval processes of working memory, emphasizing cognitive flexibility in test performance. The use of the correct word-pairs as a measure of flexibility also increases task demands for flexibility, most likely because the score is based only on successful alternation between the two different items of information. Therefore, the present study showed evidence that the modified switching verbal fluency can be a simple variation of the most used versions of fluency tests, but with greater specificity in examination of executive functions.

The use of verbal fluency tests for specific assessment of executive functions would be problematic, given the multifactorial nature of these tests. There is a common misunderstanding on tests of executive functions and "frontal lobe tests".^34^ Executive functions are closely related to frontal lobes,^35^ but also depend on the structural and functional integrity and connectivity of other regions, including the basal ganglia, the parietal lobe and the cerebellum.^36^ These processes depend on various cognitive modules that although anatomically segregated, are strongly interconnected.^37^ Similarly, the frontal lobes are involved in a number of non-executive processes, including the formation and consolidation of memories^38^ and language.^39^ In this study, an alternate verbal fluency task was used involving both of the categories previously employed in the semantic verbal fluency test (animals and fruits), increasing the focus of the particular task for the assessment of cognitive flexibility while reducing the involvement of other cognitive processes. In this context, the traditional tests of verbal fluency could be classified as more general "frontal lobe tests," while the switching versions are candidates for tests of executive functions, particularly for the cognitive flexibility component.

The present study has some limitations. The sample size is relatively small, although sufficient for the detection of moderate-large effects, which may bias the analysis for the detection of small-moderate effects. Thus, the study does not provide sufficient sample power for the detection of more discrete effect sizes. We studied a relatively homogeneous convenience sample of healthy adults and used no objective inclusion criteria (such as screening tests) to previously determine the participant cognitive status, limiting generalizability of the results to other populations or clinical groups.

An association was found between the correct word-pair produced in a modified version of the switching fluency test and cognitive flexibility. This association remained significant even after controlling for demographic factors, psychiatric symptoms, semantic knowledge and processing speed. The association of cognitive flexibility with the scores of traditional versions of verbal fluency was lower compared to this procedure. Further studies should test the validity of the method for assessing cognitive flexibility employing different approaches, including clinical studies.
